# Umbilical cord blood plasma-derived exosomes as a novel therapy to reverse liver fibrosis

**DOI:** 10.1186/s13287-021-02641-x

**Published:** 2021-11-12

**Authors:** Yu-Jen Huang, Jerry Cao, Chih-Yuan Lee, Yao-Ming Wu

**Affiliations:** 1 Department of Surgery, National Taiwan University Hospital, Taipei, Taiwan; 2 Department of Surgery, Wollongong Hospital, Loftus Street, Wollongong, NSW 2500 Australia; 3 Department of Surgery, National Taiwan University Hospital and National Taiwan University College of Medicine, No. 7, Chung-Shan South Road, Taipei, Taiwan

**Keywords:** Cirrhosis, Exosomes, Hepatic stellate cells, Inhibitor of DNA binding 1

## Abstract

**Background:**

Cirrhosis is a chronic liver disease whereby scar tissue replaces healthy liver parenchyma, leading to disruption of the liver architecture and hepatic dysfunction. Currently, there is no effective disease-modifying therapy for liver fibrosis. Recently, our group demonstrated that human umbilical cord blood (UCB) plasma possesses therapeutic effects in a rat model of acute liver failure.

**Methods:**

In the current study, we tested whether exosomes (Exo) existed in UCB plasma and if they produced any antifibrotic benefits in a liver fibrosis model.

**Results:**

Our results showed that UCB-Exo improved liver function and increased matrix metalloproteinase/tissue inhibitor of metalloproteinase degradation to reduce the degree of fibrosis. Moreover, UCB-Exo were found to suppress hepatic stellate cell (HSC) activity in vitro. These effects were associated with suppression of transforming growth factor-β/inhibitor of DNA binding 1 signaling.

**Conclusions:**

These results further support that UCB-Exo have antifibrotic effects in mice with liver fibrosis and activated HSCs and may herald a new cell-free antifibrotic therapy.

## Introduction

Liver fibrosis is a healing process associated with chronic inflammation. It may lead to cirrhosis and eventually can develop into cancer. Fibrogenesis is a vicious cycle driven by a number of damaging factors including inflammation and oxidative stress that underlie various liver conditions, such as hepatitis B and C, alcoholic liver disease, non-alcoholic fatty liver disease (NAFLD), and autoimmune liver diseases [1]. Hepatic stellate cells (HSCs) play a central role in liver fibrosis. When parenchymal epithelial cells such as hepatocytes and cholangiocytes suffer chronic injury, they secrete inflammatory mediators to promote HSC activation. This results in HSC proliferation and morphological transition to become myofibroblast-like cells, which elaborate excessive extracellular matrix (ECM) that accumulates in the liver to disrupt hepatic architecture and lead to liver dysfunction [2, 3]. As a result, termination of this vicious cycle or elimination of fibrogenic HSCs may represent an approach for future antifibrotic therapies in the clinical setting of liver cirrhosis. During the vicious cycle, TGF-β (transforming growth factor β), a profibrotic cytokine, contributes to liver fibrosis through phosphorylation of Smad 2 to activate HSC differentiation into myofibroblasts [4, 5]. Therefore, enhanced TGF-β signaling in liver fibrosis correlates with progression of fibrosis [6]. Recent research indicates that TGF-β may interact with inhibitor of DNA binding protein (ID) 1–4-associated signaling pathways. ID belongs to the helix–loop–helix (HLH) protein family, which is responsible for cell cycle regulation and cell differentiation [7]. Of all the ID proteins, ID1 is linked to tumorigenesis, cellular senescence, and cell proliferation [8]. In addition, ID1 levels are highly expressed in hepatocellular carcinoma [9, 10]. Therefore, inhibition of ID1 expression may be a promising therapeutic approach.

Stem cell therapy has been showing promising effects in regenerative medicine. In general, stem cells have the capacity to differentiate into diverse cell types to replace the damaged cells at a site of injury. Moreover, stem cells secrete biological factors to affect surrounding tissues [11]. Although stem cell therapy provides hope for degenerative diseases, there still exist some challenges, including host cell rejection, phenotypic stability, and tumor risk [12, 13]. This calls for safe and effective cell-free therapies. Recent research has focused on exosomes as one such therapeutic agent. Exosomes are produced by various cells, such as stem cells, cancer cells, and immune cells. In addition, exosomes are present in various body fluids, including blood, urine, plasma, amniotic fluid, and semen. In fact, exosomes are responsible for cell-to-cell communication and signal transmission, as well as facilitating repair of damaged tissues. Umbilical cord blood (UCB) contains mesenchymal stem cells (MSCs) and hematopoietic stem cells, and the proliferation and function of these stem cells are affected by a variety of cytokines, growth factors, and immunomodulatory agents [14, 15]. These factors are reported to improve functional performance and reduce structural damage in a mouse model of acute ischemic stroke and have been suggested as potential cell-free therapeutic agents [16]. However, the therapeutic potential of UCB plasma has not been elucidated in liver diseases, and a recent study has shown that exosomes recapitulate the therapeutic effects of stem cell therapy [17, 18]. Our previous research demonstrated that UCB plasma biomolecules decrease the damage in d-galactosamine (d-GalN)-induced acute liver failure [19]. Hence, our current work aims to characterize UCB plasma-derived exosomes and demonstrate their potential as a new promising tool in the cell-free therapeutic armamentarium for the treatment of liver fibrosis.

## Materials and methods

### Isolation and characterization of exosomes

Human UCB and peripheral blood (PB) plasma were purchased from AllCells LLC (Alameda, CA), and exosomes were acquired using ExoQuick™ Exosome isolation reagent (System Bioscience Inc., Mountain View, CA). Briefly, plasma was mixed with the exosome isolation reagent and incubated at 4℃ overnight. The mixture was centrifuged at 1500 g for 5 min, then the supernatant was removed. The pellet was washed with PBS and centrifuged. Exosome fractions were characterized with CD63, CD9, CD81, and Hsp70 by the ExoAB Antibody kit (System Bioscience Inc.). Exosome sizes and concentrations were quantified using NanoSight (NanoSight Ltd., Amesbury, UK, Malvern. Com). Protein concentrations were determined by Pierce BCA assay (Thermo Scientific, Rockford, USA). The morphology of exosomes was determined by transmission electron microscopy (TEM). Exosomes were mounted on copper grids and were added 2% of uranyl acetate solution for 30 s. The copper grids were then moved to the sample plate and stored in drying oven. HT-7700 Hitachi TEM was used to image exosome samples at a voltage of 100 kV. For exosome staining and uptake, exosomes were labeled with PKH-67 Green Fluorescent Cell Linker Kit for General Cell Membrane Labelling (Sigma) according to the manufacturer’s protocol. Briefly, the exosomes were diluted in PBS and added to Diluent C (1 mL Diluent C with same volume of PBS as control), then 4 μL of PKH-67 were added to it and incubated for 4 min. This was followed by addition of 2 mL of 1% BSA (Sigma) to bind the excess dye. This was washed 4 times with 5 mL of PBS using a 300-kDa filter (Microcon TM-300, Millipore) to remove excess dye, and then incubated with HSCs for 24 h. Uptake efficiency was observed with flow cytometry. Exosome-free PBS receiving the same treatment was used as control.

### Protein extraction and gel electrophoresis

The proteins were extracted with ExtractPRO™ Protein extraction reagent (Visual Protein, Taiwan). The protein was analyzed using 12.5% SDS–PAGE. After electrophoresis, the gels were stained with VisPRO 5 min Protein Stain Kit (Visual Protein, Taiwan). The gel lanes corresponding to the samples were cut in 2 slices, and each slice was processed for in-gel digestion according to the method of Shevchenko. Briefly, slices were washed/rehydrated three times in 25 mM ABC (ammonium bicarbonate pH 7.9) + 50% ACN (acetonitrile)/50 mM ABC (ammonium bicarbonate pH 7.9). Subsequently, proteins were reduced with 10 mM dithiothreitol for 1 h at 56 °C and alkylated with 25 mM iodoacetamide for 45 min at 24 °C in the dark. After two subsequent wash cycles, the slices were dried and incubated overnight with 20 ng/μL MS-grade Trypsin Gold (Promega, Madison, WI). Peptides were extracted three times in 10 μL of 50% ACN in 1% formic acid. The volume was dried out in a vacuum centrifuge prior to LC–MS/MS analysis.

### Nano-LC separation and mass spectrometry

Peptides were separated using an Ultimate 3000 nano-LC system (Thermo Fisher Scientific, Bremen, Germany). Mobile phase A was 0.1% formic acid in water, and mobile phase B was composed of 100% acetonitrile with 0.1% formic acid. A segmented gradient in 90 min from 2 to 35% solvent B at a flow rate of 300 nL/min and a column temperature of 35 °C were used. Intact peptide mass spectra and fragmentation spectra were acquired on a Thermo Scientific™ Orbitrap Fusion™ Lumos™ Tribrid™ Mass Spectrometer (Thermo Fisher Scientific, UK). Mass spectrometry analysis was performed in a data-dependent mode with Full-MS (externally calibrated to a mass accuracy of < 5 ppm, and a resolution of 120,000 at *m*/*z* = 200) followed by HCD-MS/MS of the most intense ions in 3 s. High-energy collision activated dissociation (HCD)-MS/MS (resolution of 15,000) was used to fragment multiply charged ions (charge state 2–7) within a 1.4 Da isolation window at a normalized collision energy of 32 eV.

### Bioinformatic analysis

The accession number of each identified protein was loaded to the FunRich software V3.1.3 (http://www.funrich.org) and mapped according to their Gene Ontology (GO), to determine their biological and functional properties. The identified proteins were compared with available exosome data from ExoCarta database (http://www.exocarta.org).

### Cells

Mouse HSCs were isolated with density gradient centrifugation using C57BL/6 mice. Briefly, the mouse liver was perfused in situ with pronase and collagenase (Sigma), followed by Histodenz (8.3%, Sigma) density gradient centrifugation. HSCs were maintained in Medium 199 (Sigma) with 10% fetal bovine serum (FBS, Hyclone Laboratories Inc, USA), antibiotics, and L-glutamine (Gibco, Carlsbad, CA) at 37 °C in a 5% CO_2_ incubator. All experiments were performed at passages 2 to 7. Human HSC line (LX2) was cultured in DMEM medium (Gibco, Carlsbad, CA) with 10% FBS, antibiotics, and L-glutamine at 37 °C in a 5% CO_2_ incubator.

Cell proliferation assay was performed using the CyQUANT® Cell proliferation assay kit (Invitrogen, USA). HSCs (5,000/well) were seeded in 96-well culture plates with Medium 199 containing 0.2% Exo-free FBS, and treated with different concentrations of UCB-Exo (10, 20, 40, 80, 160 μg/mL). After 24 h, the medium was removed, washed once with PBS, and then stored at -80 °C. On the next day, it was added to a detection reagent for 5 min and then read with Ex480/Em520. To determine LX2 cell proliferation, the LX2 cells were seeded in 96-well culture plates and added to 20 μg/mL (~ 5 × 10^6^ particles) of UCB-Exo or PB-Exo with/without TGF-β (5 ng/mL). After 24 h, cell proliferation was measured. For TGF-β stimulation, LX2 cells were pretreated with UCB-Exo or PB-Exo for 24 h, then stimulated by TGF-β (5 ng/mL) for 0, 0.5, 1, and 2 h. Cells were then collected for western blotting.

### CCl_4_-induced liver fibrosis and UCB-Exo therapy

C57BL/6 mice (7-week-old males) received intraperitoneal injections of carbon tetrachloride (CCl_4_, 1 μg/g body weight, prepared in olive oil, 1:5; Sigma) twice per week. After one month of CCl_4_ challenge, the mice were randomly divided into two groups (*n* = 10/group). One group was infused with PBS, while the other group was infused with UCB-Exo (250 μg/0.1 mL) via a tail vein twice a week. Both groups received sustained administrations of CCl_4_. The mice were sacrificed for analysis of liver tissue and serum at the second and third months after CCl_4_ injection (Fig. 3A). All experimental procedures involving animals were approved by the Institutional Animal Care and Use Committee of National Taiwan University. All animals were purchased from National Laboratory Animal Center, Taipei, Taiwan.

### Liver enzyme, hepatic collagen content, and gelatinase activity measurements

Serum aspartate aminotransferase (AST) and alanine aminotransferase (ALT) were measured with FUJI DRI-CHEM slide (FUJIFILM Drichen 4200, Japan). Hepatic collagen content was determined by Sircol Collagen Assay (Biocolor, Belfast, Northern Ireland). Collagen concentrations were calculated using a standard curve generated by acid-soluble type 1 collagen and expressed relative to total protein by Pierce BCA measurement (Thermo Scientific, Rockford, USA). For gelatinase (MMP-2 and MMP-9) activity measurements, InnoZymeTM Gelatinase (MMP-2/MMP-9) activity assay kit (Millipore) was used according to manufacturer’s instructions.

### Histopathological and immunohistochemical staining

Liver tissue was paraffin-embedded, processed into 5 μm sections, and then stained with hematoxylin and eosin (H&E) (Sigma) and Picro-Sirius red solution (ScyTek Laboratories, Logan, USA). Fibrosis score was quantified with reference to the Ishak fibrosis stage system [20]. The mean value of 10 randomly selected areas per mouse, magnification × 100, was obtained. For immunohistochemical staining, liver sections were incubated with diluted primary antibodies against anti-αSMA (1:500; Sigma) and inhibitor of DNA binding 1 (ID1, 1:500; Santa Cruz Biotechnology, Inc, Oregon, USA), according to the manufacturer’s instructions. The proteins were visualized by EnVision + Dual Link System-HRP (DAB +), and counter-stained with Mayer’s Hematoxylin (Dako, Carpinteria, CA, USA).

### Western blot

Mouse liver tissue was homogenized in lysis buffer (Sigma) supplemented with proteinase inhibitor cocktail (Roche Diagnostics, Germany), boiled in SDS protein buffer (Protech, Taiwan), and separated by 10% of SDS–PAGE following transfer to PVDF membrane. Primary antibodies were targeting TGF-β, MMP-2, MMP-9, MMP-13, TIMP-1, TIMP-2 (purchased from Abcam, Cambridge, MA), collagen type I, α-SMA (Sigma), ID1 (Santa Cruz Biotechnology, Inc, Oregon, USA), Smad2/3, and phospho-Smad2/3 (Cell Signaling Transduction). Secondary antibodies were anti-rabbit and anti-mouse antibodies (IgG-HRP, Jackson ImmunoResearch, Dianova, Hamburg). Proteins were visualized by enhanced chemiluminescence detection (ECL, Millipore Corporation, Billerica, MA, USA). ImageJ software was used for quantification.

### Quantitative PCR

Total RNA was extracted with TRIzol Reagent (Invitrogen, Carlsbad, CA, USA) using Direct-Zol RNA MiniPrep kit (Zymo Research, Orange, CA, USA). 1 µg samples of RNA were reverse transcribed using a RevertAid^TM^ H Minus First Strand cDNA Synthesis Kit (Fermentas, Thermo Scientific, Schwerte, Germany). RT-PCR was performed with Luminaris color HiGreen qPCR Master Mix (Fermentas, Thermo Scientific) on a CFX Connect Real-Time System (Bio-Rad, Hercules, CA, USA). The primer sequences are listed in Table 1.

**Table 1:** Primer sequences

Target gene	Primer sequence (5–3′)	Amplicon size (bp)
MMP-13	For: AACCCTAAGCACCCCAAAACA Rev: GGTCAAAAACAGTTCAGGCTCAA	150 bp
Pro-Collagen type I-α1	For: CCAGCGGTGGTTATGACTTCA Rev: GCTGCGGATGTTCTCAATCTG	167 bp
MMP-2	For: ACCATGCGGAAGCCAAGAT Rev: GCCCGAGCAAAAGCATCAT	155 bp
MMP-9	For: CCTACTGCTGGTCCTTCTG Rev: GGCTTCCTCCGTGATTCG	157 bp
TIMP-1	For: CATGGAAAGCCTCTGTGGAT Rev: CTCAGAGTACGCCAGGGAAC	132 bp
TIMP-2	For: CACAGACTTCAGCGAATGGA Rev: CCAGCATGAGACCTCACAGA	124 bp
TGF-β	For: TTGCCCTCTACAACCAACACAA Rev: GGCTTGCGACCCACGTAGTA	103 bp

### Statistical analysis

Data are presented as mean ± S.E.M. and are compared using two-tailed Student’s *t* test or one-way analysis of variance with Turkey’s post hoc test used for multiple comparisons. Statistical significance is set to *p* < 0.05.

## Results

### Isolation and identification of exosomes derived from UCB and PB

**Fig. 1:**
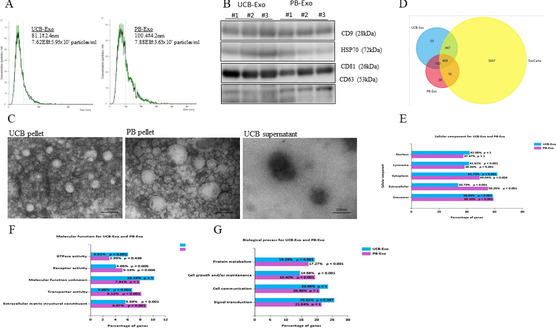
Exosome identification and proteomic analysis in UCB-Exo and PB-Exo. **A** Size distribution of exosomes as measured by nanoparticle tracking analysis (NTA) showed that the UCB-Exo particle was 80.1 nm and concentration was 7.62 × 108 particles/mL, and the PB-Exo particle was 100.4 nm and concentration was 7.88 × 108 particles/mL. **B** Western blotting assay revealed that UCB-Exo and PB-Exo were positive for CD9, HSP70, CD81, and CD63. **C** TEM images of exosomes isolated from UCB and PB show spherically shaped particles. Bioinformatic analysis of UCB-Exo and PB-Exo. A Venn diagram of UCB-Exo and PB-Exo against ExoCarta (**D**). GO analysis for cellular components (**E**), molecular function (**F**), and biological processes of UCB-Exo and PB-Exo (**G**)

Our previous experiments showed that UCB contains a variety of cytokines with therapeutic effects in the acute liver failure rat model. Recently, exosomes have been shown to participate in cell communication and bring hope for therapeutic opportunities. Therefore, we believed that UCB may contain exosomes that could be used as therapeutic agents. First, we obtained the vesicles from UCB and PB with ExoQuick reagent. Nanoparticle tracking analysis showed that the size of the vesicles ranged from 52.0 to 126.0 nm in diameter and the median size of the vesicles was 81.1 ± 2.4 nm for UCB-Exo (Fig. 1A). For PB-Exo, the size of vesicles ranged from 56.2 to 153.5 nm in diameter and the median size was 100.4 ± 4.2 nm (Fig. 1A). They both were similar to the reported sizes of exosomes [21]. The UCB- and PB-Exo average particle count was 7.62 × 10^8^ and 7.88 × 10^8^ per mL, respectively. The membrane integrity of exosomes was confirmed in the TEM image and appeared spherical in structure after isolation from UCB and PB (Fig. 1C). The particle sizes were consistent in samples isolated from UCB-Exo and PB-Exo under TEM. Next, to further confirm that our preparation contained exosomes, we used western blotting to detect four common exosome markers, including CD63, CD9, CD81, and HSP70. Our data clearly verify that UCB- and PB-Exo were positive for these exosome markers (Fig. 1B). These results indicated that exosomes exist in UCB and PB.

### Bioinformatic analysis of exosomes from UCB and PB

Proteomic analysis of the different sources of exosomes revealed 1,011 and 555 proteins were present in UCB-Exo and PB-Exo, respectively. About 80% of UCB-Exo and PB-Exo proteins matched ExoCarta, indicating that the results were remarkably reliable (Fig. 1D). Comparing UCB-Exo and PB-Exo using a Venn diagram revealed 409 common proteins, and 53 and 28 proteins being exclusive to UCB-Exo and PB-Exo, respectively (Fig. 1D). GO analysis of the two source-derived exosomes showed that 60% of their component proteins could be correlated to available exosome data from ExoCarta. The exosomal proteins derived from UCB are more closely related to the proteins in the cytoplasm than the exosomal proteins derived from PB (Fig. 1E). In terms of molecular function, these proteins played significant roles in ECM, transporter activity, and GTPase activity (Fig. 1F). These proteins were also key participants in biological processes including protein metabolism and cell growth (Fig. 1G). These results suggest that plasma-derived exosomes have the potential for regeneration through these candidate proteins.

### UCB-Exo treatment inhibits HSC proliferation and reduces collagen production

**Fig. 2:**
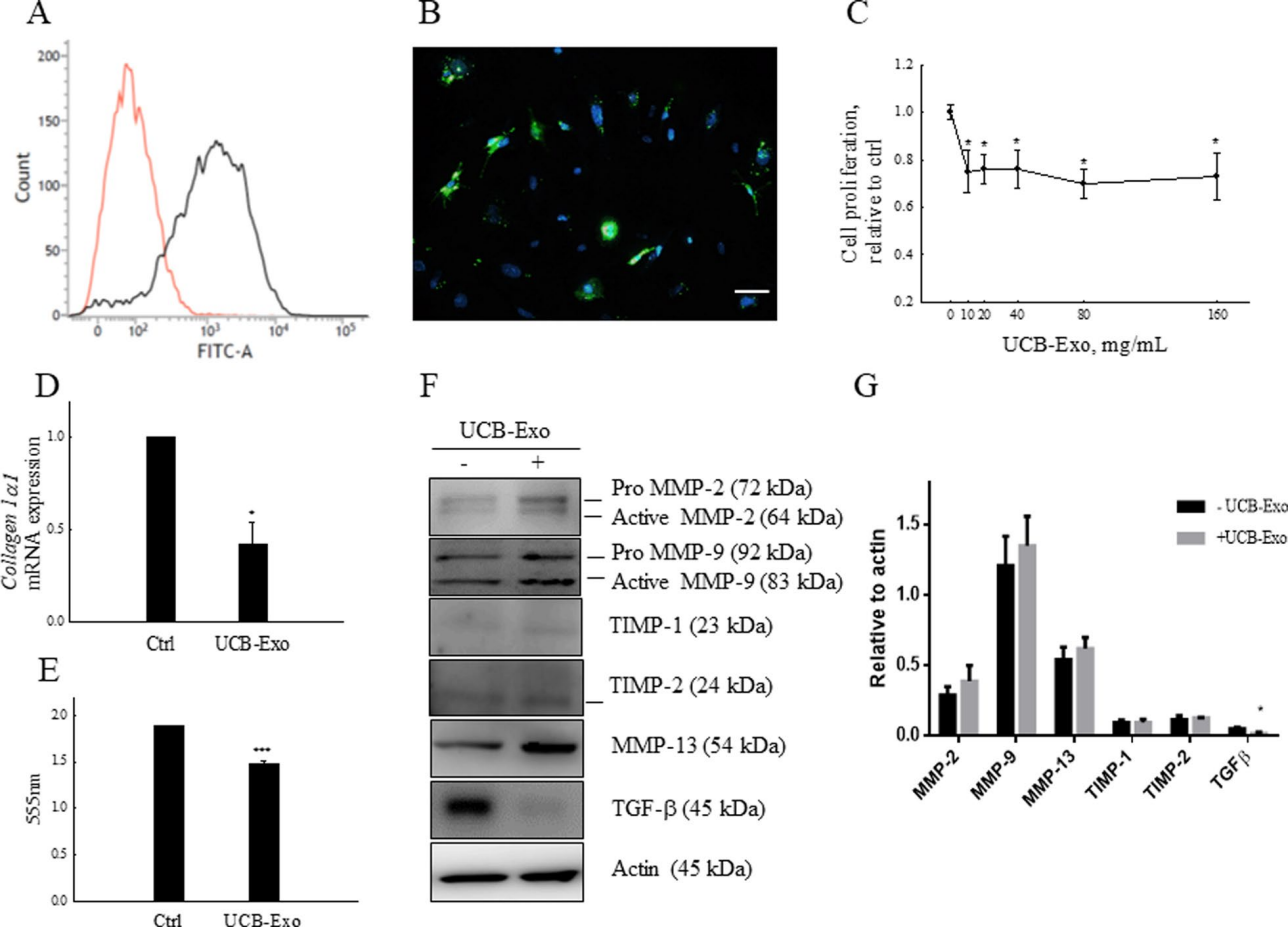
UCB-Exo inhibit primary HSC activation and collagen production. Primary HSCs were incubated with PKH-labeled exosomes (20 µg/mL) for 12 h and analyzed by flow cytometry (**A**) and microscopy (**B**). Blue: DAPI; Green: PKH-labeled UCB-Exo. Scale bar: 50 µm. HSCs were exposed to different concentrations of UCB-Exo for 24 h and cell proliferation is presented in (**C**). qRT-PCR analyses of collagen 1α1 expression in treatment with UCB-Exo for 24 h (**D**). Soluble collagen I content was measured in culture media after 24 h of UCB-Exo treatment (**E**). Western blotting analysis of MMP-2, MMP-9, MMP-13, TIMP-1, TIMP-2, and TGF-β expression after 72 h of UCB-Exo treatment (**F**). Quantification of western blot results was performed by calculating the ratio of the value to that of actin. Data are presented as the mean ± SEM. *p < 0.05 and ***p < 0.001

HSCs are major ECM producers in the liver, hence we prioritized testing whether UCB-Exo could be taken up by HSCs. Flow cytometry results showed that 78% of UCB-Exo labeled with PKH-67 were taken up by HSCs (Fig. 2A), and these were localized in the perinuclear region (Fig. 2B). Next, to evaluate the effect of UCB-Exo on cell proliferation of activated HSCs, activated HSCs were treated with different concentrations of UCB-Exo for 24 h. Results showed that cell proliferation was significantly decreased starting from 10 μg/mL of UCB-Exo. Surprisingly, increasing UCB-Exo concentration further did not affect cell proliferation (Fig. 2C). Therefore, we chose the concentration of 20 μg/mL of UCB-Exo to perform further experiments. It is known that activated HSCs are the major cell type responsible for collagen production [22–24]. Therefore, we examined whether collagen production in HSCs would be affected by UCB-Exo. After treatment with 20 μg/mL of UCB-Exo for 24 h, QPCR results showed that collagen 1α2 mRNA levels were significantly decreased in activated HSCs (Fig. 2D). In the meantime, measurements of culture media demonstrated a reduction in soluble collagen I content in activated HSCs treated with UCB-Exo (Fig. 2E). Moreover, ECM remodeling was related to MMP expression and associated with TGF-β [25]. As shown in Fig. 2F, G, western blotting indicated that TGF-β levels were significantly decreased in activated HSCs treated with UCB-Exo. In addition, MMP-2, MMP-9, and MMP-13 protein expressions were increased after UCB-Exo treatment for 72 h in activated HSCs. TIMP-1 and TIMP-2 expressions, which are inhibitors of MMP-9 and MMP-2, were not significantly different. Therefore, UCB-Exo treatment may significantly reduce cell proliferation, and enhance MMP levels to decrease collagen production.

### UCB-Exo improve liver function and decrease damage from CCl_4_

**Fig. 3:**
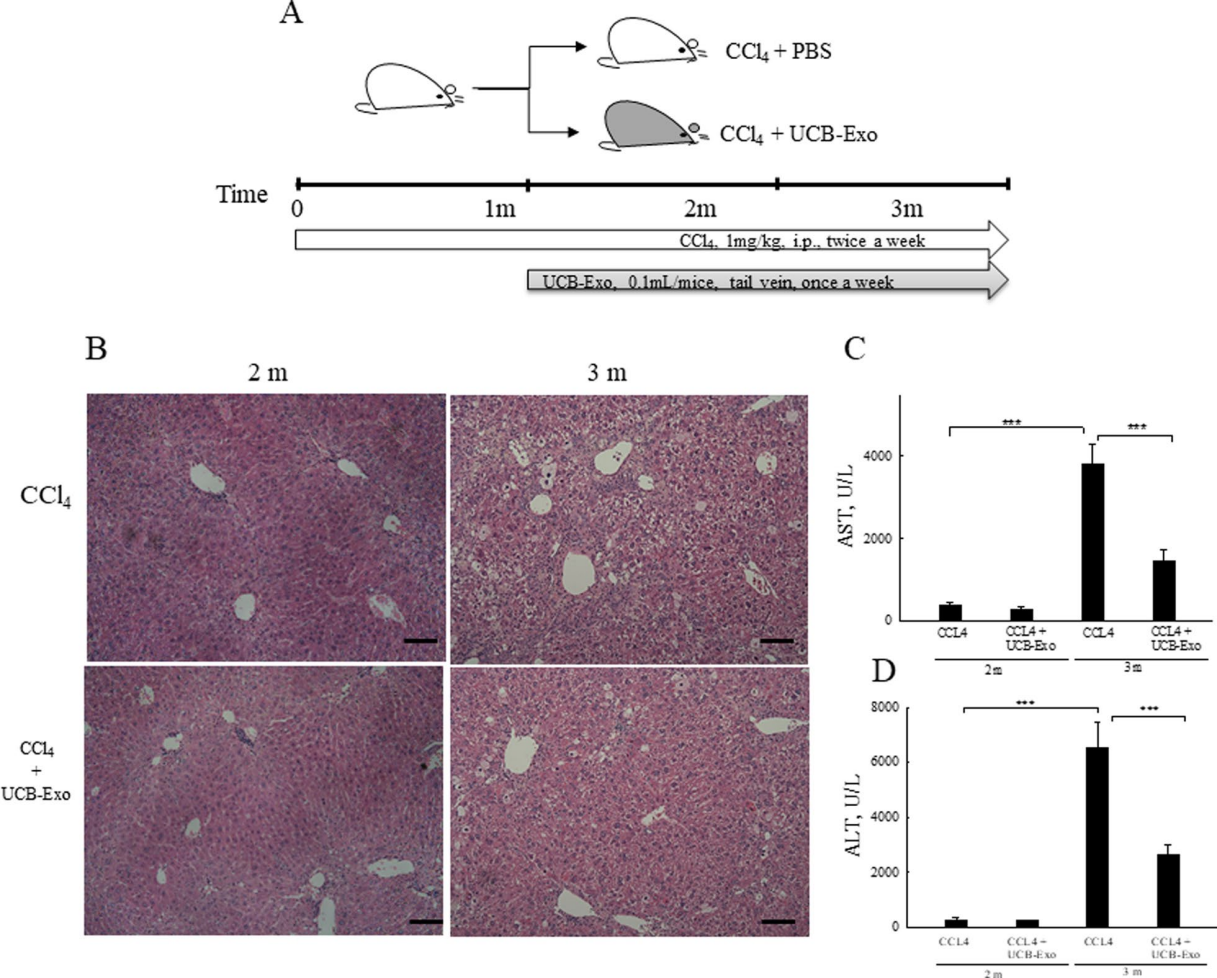
UCB-Exo reduce histological injuries and improve liver function in mice with CCl_4_-induced liver fibrosis. Schematic diagram of liver fibrosis. Mice were administered CCl_4_ for 1 month (*n* = 20) and divided into two groups, namely, a CCl_4_ group (CCl_4_ + PBS), and an UCB-Exo group (CCl_4_ + UCB-Exo for 2 months) (**A**). Liver sections were stained with H&E and are shown in (**B**). Scale bar: 100 μm. Serum AST (**C**) and ALT (**D**) levels were analyzed. Data are presented as the mean ± SEM. ***p < 0.001

Next, we investigated the therapeutic efficacy of UCB-Exo in liver fibrosis using the CCl_4_-induced liver fibrosis mouse model. The administration of CCl_4_ for 3 months caused severe liver damage as characterized by significantly elevated levels of serum AST (2 months: 372.4 ± 70.6 U/L; 3 months: 3818.0 ± 471.2 U/L; Fig. 3C) and ALT (2 months: 272.6 ± 68.0 U/L; 3 months: 6524.0 ± 928.7 U/L; Fig. 3D), and as supported by the histopathological examination. H&E staining demonstrated disruption of liver architecture, balloon cells, and inflammatory cell infiltration after 3 months of CCl_4_ exposure (Fig. 3B). However, no mice died during this experiment (prior to sacrifice). After receiving CCl_4_ for 1 month, the mice were infused with UCB-Exo 4 times (once a week). Histopathological examination (Fig. 3B) and serum levels of AST and ALT (Fig. 3C, D) showed no significant change when compared to the CCl_4_ groups. However, when receiving 8 infusions of UCB-Exo, the results showed that AST (1472.5 ± 271.9 U/L; Fig. 3C) and ALT (2651.3 ± 364.5 U/L; Fig. 3D) levels were significantly decreased compared to the group exposed to CCl_4_ for 3 months. Altogether, incidence and severity of histopathological lesions were reduced in the group receiving UCB-Exo infusion for 8 times, but these were not significantly different between the 4 times group and the CCl_4_ group (Fig. 3B).

**Fig. 4:**
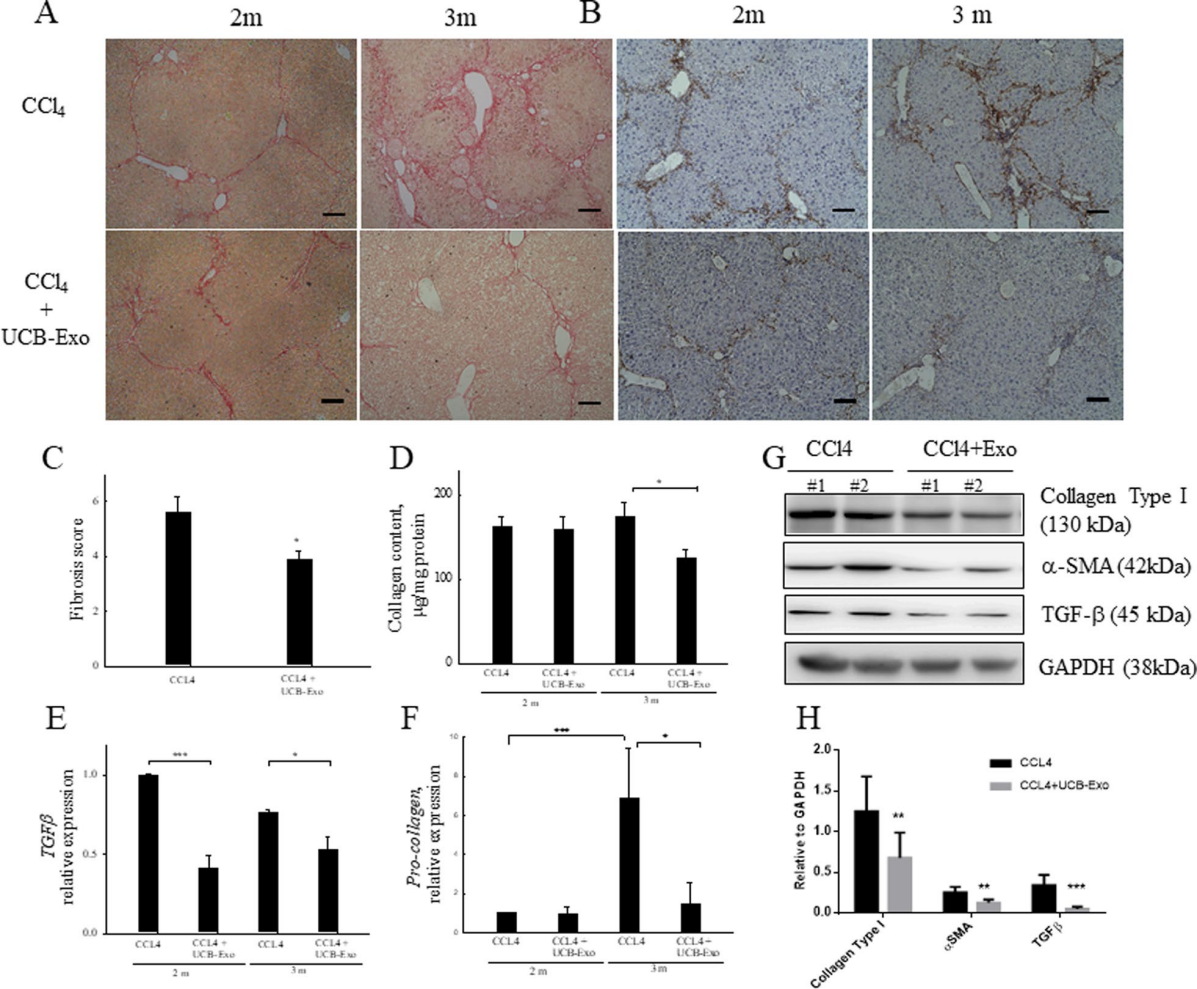
UCB-Exo attenuate CCl_4_-induced liver fibrosis and reduce collagen deposition. Liver sections were stained with Sirius red (**A**) and the Sirius red-positive area (red color) was determined by Ishak method (**C**). Immunohistochemical staining of liver sections for α-SMA (brown color) (**B**) in CCl_4_ and CCl_4_ + UCB-Exo groups. Collagen contents were measured in liver tissue of CCl_4_ and CCl_4_ + UCB-Exo groups (**D**). qRT-PCR analyses of TGF-β (**E**) and pro-collagen (**F**) expressions in liver tissue of CCl_4_ and CCl_4_ + UCB-Exo groups. Western blotting analysis of collagen type I, α-SMA, and TGF-β expressions in liver tissue of CCl_4_ and CCl_4_ + UCB-Exo groups (**G**). Quantification of western blot results was performed by calculating the ratio of the GAPDH. Data are presented as the mean ± SEM. Scale bar: 100 μm. *p < 0.05 and ***p < 0.001

Subsequently, we investigated whether UCB-Exo could decrease collagen production. Sirius red staining may be used to visualize the collagen network and determine the degree of fibrosis in tissue. As Fig. 4A shows, the fibrotic area was significantly decreased in the liver that was infused with UCB-Exo for 8 times. Based on the results of Sirius red staining, we referred to the Ishak fibrosis grading system [26] to determine the histological fibrosis score ranging from 0 (no fibrosis) to 6 (cirrhosis). The average fibrotic scores were significantly lower in the UCB-Exo group compared to the CCl_4_ group (3.88 ± 0.29 vs. 5.6 ± 0.55) (Fig. 4C). In addition, soluble collagen content was significantly lower in the liver tissue of UCB-Exo mice compared to CCl_4_ mice at 3 months (Fig. 4E). Examination of collagen expression using QPCR (Fig. 4F) and western blotting (Fig. 4G, H) revealed that levels of pro-collagen mRNA and protein levels were significantly decreased after 8 times of UCB-Exo administration. Moreover, activated HSCs were not only responsible for collagen production, but also highly expressed α-SMA and secreted TGF-β. Immunohistochemical analysis of α-SMA showed that the group infused with UCB-Exo 8 times possessed significantly less α-SMA positive areas than the CCl_4_ group, with the former group demonstrating diminished fibrotic networks (Fig. 4B). Further results showed that expression of α-SMA protein was reduced in the 8-time UCB-Exo infusion group. QPCR assay (Fig. 4D) and western blotting (Fig. 4G, H) indicated that TGF-β expression could be significantly lower in 4-time UCB-Exo infusion group compared to the CCl_4_ group at 2 months. Therefore, these results imply that UCB-Exo may exert an antifibrotic effect by decreasing collagen deposition.

### UCB-Exo exert antifibrotic effects in the mouse model of liver fibrosis

**Fig. 5:**
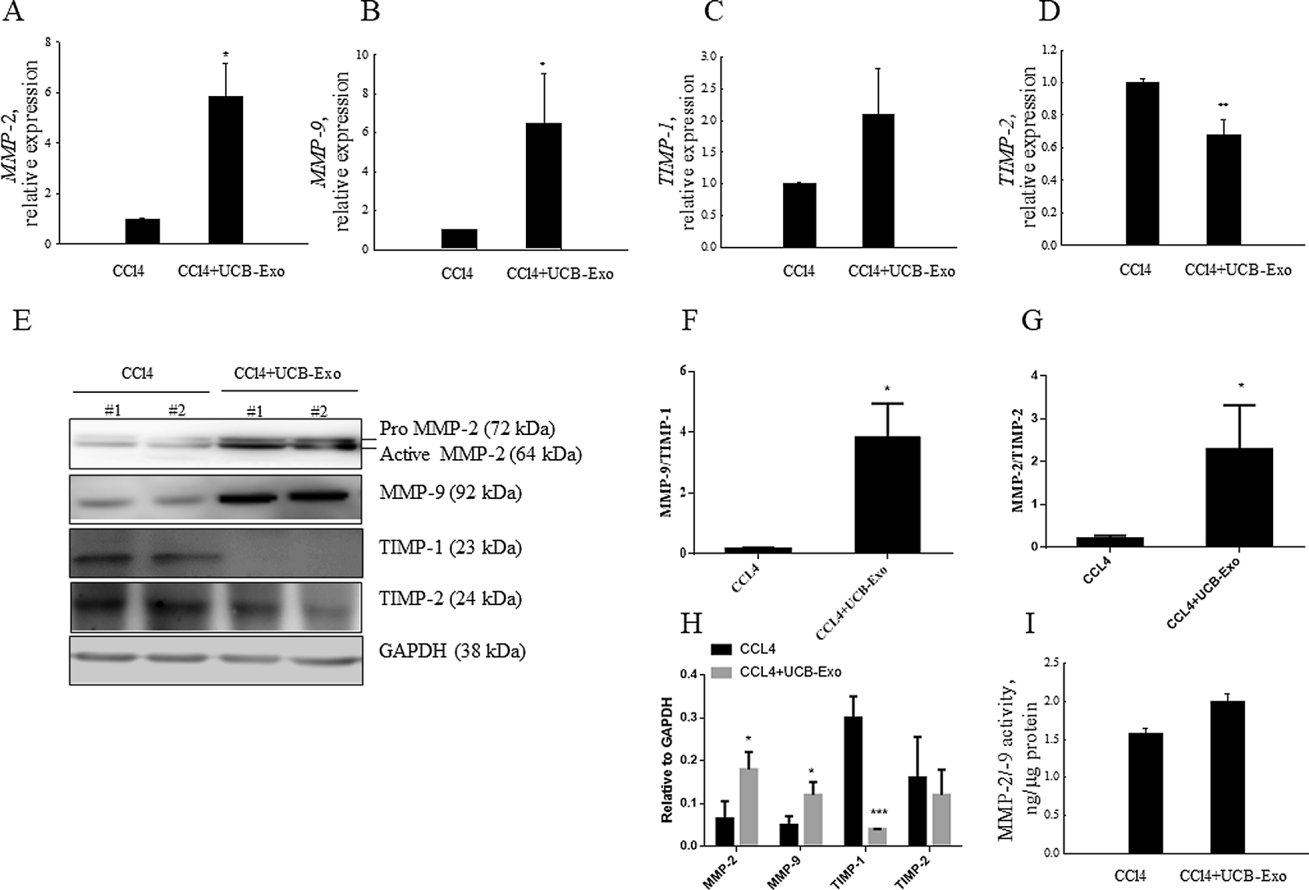
UCB-Exo increase MMP degradation in CCl_4_-induced liver fibrosis. qRT-PCR analyses of MMP-2 (**A**), MMP-9 (**B**), TIMP-1 (**C**), and TIMP-2 (**D**) expressions after 2 months of UCB-Exo treatment and in the CCl_4_ group. Western blotting assay for MMP-2, MMP-9, TIMP-1, and TIMP-2 in the liver tissue of CCl_4_ and CCl_4_ + UCB-Exo groups (**E**). Quantification of western blot results was performed by calculating the ratio of MMP-9 to TIMP-1 (**F**), the ratio of MMP-2 to TIMP-2 (**G**), and the ratio of the value to GAPDH (**H**). MMP-2 and MMP-9 activities were increased in the liver tissue of the CCl_4_ + UCB-Exo group compared to the CCl_4_ group in the third month (I). Data are presented as the mean ± SEM. *p < 0.05, **p < 0.01 and ***p < 0.001

During the fibrogenesis, activated HSCs produce ECM that accumulate in the liver. ECM homeostasis is regulated by the balance of MMPs and TIMPs, which is important in tissue remodeling and liver fibrogenesis. MMP-2 is responsible for turnover of ECM and tissue homeostasis, whereas MMP-9 is involved in tissue injury and fibrogenesis. Their activity is inhibited by TIMP-2 and TIMP-1, respectively [27]. We measured MMP-2, MMP-9, and their inhibitors (TIMP-1, TIMP-2) in the CCl_4_-induced liver fibrosis group with/without UCB-Exo treatment. Figure 5 shows that mRNA and protein expression of MMP-2 and MMP-9 were significantly higher in the UCB-Exo 8-time infusion group (Fig. 5A, B). Although TIMP-1 mRNA was increased in the 8-time UCB-Exo infusion group, this did not reach statistical significance (Fig. 5C), whereas protein expression of TIMP-1 was inhibited by treatment with UCB-Exo for 8 times (Fig. 5E, H). Similarly, TIMP-2 mRNA and protein levels were also significantly lower (Fig. 5D, E, H). Besides, the activity of MMP-2/9 was slightly increased in the UCB-Exo group in comparison with the CCl_4_ group (Fig. 5I, 1.57 ± 0.08 vs 2.0 ± 0.10 ng/μg protein). The net activity of ECM turnover or fibrosis regression would depend on the balance between MMPs and TIMPs [28]. Therefore, we performed quantitative analysis of western blot results and assessed the ratios of MMP-2/TIMP-2 and MMP-9/TIMP-1. Our results indicated a threefold increase in proteolytic activity for the UCB-Exo group in comparison with the CCl_4_ group (Fig. 5F, G). These results revealed that UCB-Exo treatment tilted the MMP/TIMP balance against fibrogenesis.

### UCB-Exo treatment hinders HSC activity through inhibition of the TGF-β-ID1 signaling pathway both in vivo and in vitro.

**Fig. 6:**
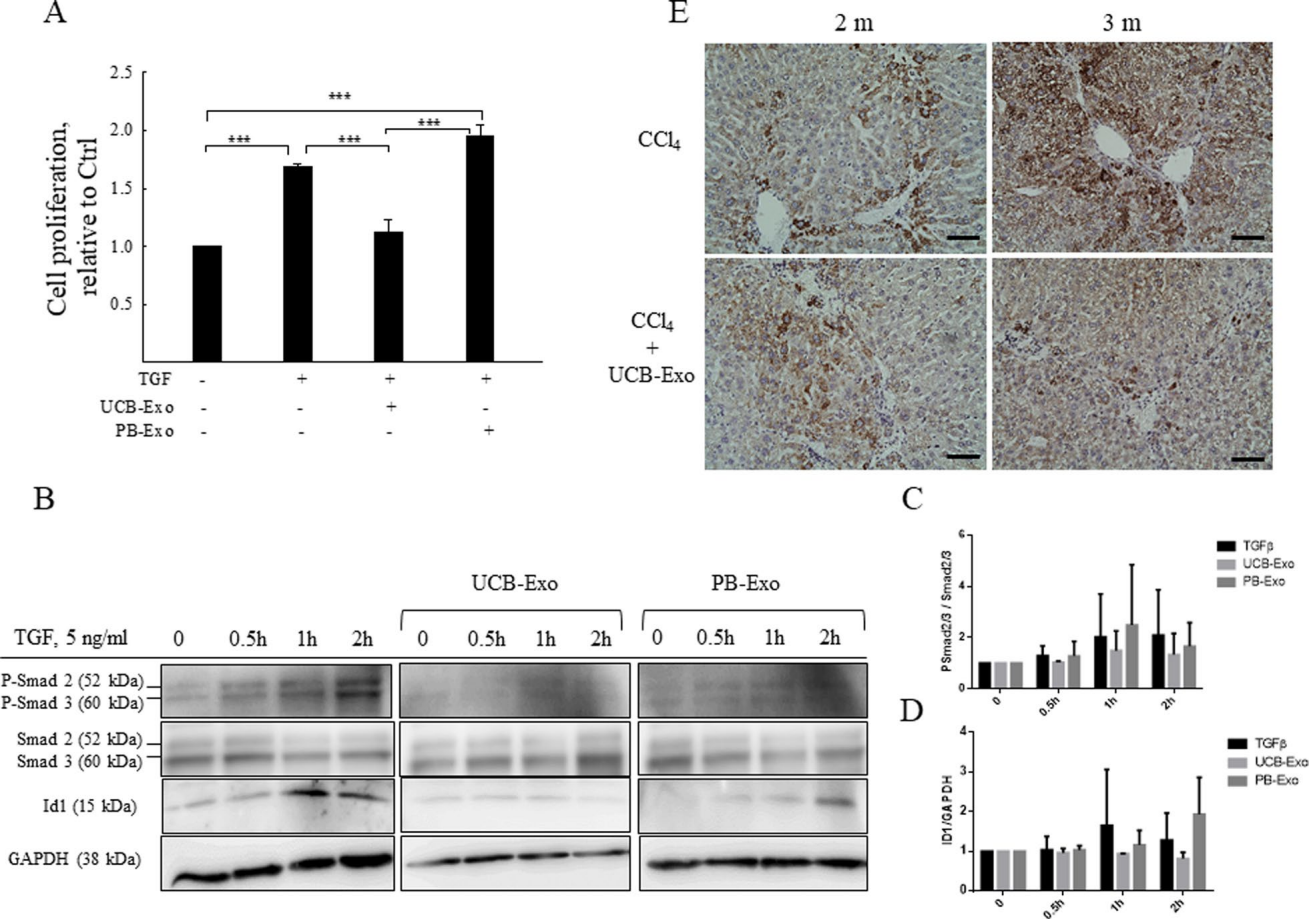
UCB-Exo inhibit cell proliferation via suppressing TGF-β/ID1 signaling. LX2 cells were treated with 20 μg/mL of UCB-Exo or PB-Exo with/without 5 ng/mL of TGF-β for 24 h and cell proliferation was determined by CyQUANT assay kit (**A**). Western blotting analysis of phospho-Smad2/3, Smad2/3, and ID1 expressions in LX2 cells pretreated with 20 μg/mL of UCB-Exo or PB-Exo for 24 h, then stimulated by TGF-β for different durations (**B**). Quantification of western blot results was performed by calculating the ratio of pSmad2/3 to Smad2/3 (**C**) and the ratio of ID1 to GAPDH (**D**). Immunohistochemical staining of liver sections for ID1 (brown color) (**E**). Data are presented as the mean ± SEM. ***p < 0.001

Typically, TGF-β is a master pro-fibrogenic cytokine that is associated with HSC activation and liver fibrosis [29]. Here, we studied whether UCB-Exo and PB-Exo exerted protective effects in LX2 cells stimulated by TGF-β. Our data showed that cell proliferation was significantly increased by 1.68-fold when treated with 5 ng/mL of TGF-β for 24 h. Furthermore, cell proliferation was decreased by 1.12-fold if treated with UCB-Exo. In contrast, with PB-Exo treatment, cell proliferation was increased by 1.95-fold (Fig. 6A). Thus, cell proliferation appeared to be suppressed by UCB-Exo treatment, but not by PB-Exo. To understand the response to TGF-β downstream, we pretreated LX2 cells with UCB-Exo or PB-Exo for 24 h, followed by stimulation with TGF-β for different durations (0, 0.5, 1, and 2 h). Western blotting showed that TGF-β stimulation could activate phospho-Smad2/3 expression depending on the duration of TGF-β exposure, with a significant increase in expression observed at 2 h duration. As Fig. 6B shows, UCB-Exo and PB-Exo slightly suppressed phospho-Smad2/3 expression in the TGF-β stimulation condition. ID1 protein expression was significantly induced by TGF-β stimulation in LX2 cells, and UCB-Exo was found to inhibit ID-1 expression substantially. Although there seemed to be a trend that UCB-Exo reduced pSmad2/3 and ID1, there was no significant difference between treatment with UCB-Exo or PB-Exo (Fig. 6C, D). Based on the verified correlation between ID1 and TGF-β in LX2 cells, we further detected the expression of ID1 in the liver fibrosis mouse model. Immunohistochemical analysis revealed that ID1 was localized to the cell cytoplasm, and it was strongly expressed in the liver sections exposed to CCl_4_. After infusion of UCB-Exo for 4 times, ID1 expression did not change significantly in comparison with the control group after exposure to CCl_4_ for 2 months. After UCB-Exo infusion for 8 times, however, ID1 expression was indeed significantly decreased compared with the control group after CCl_4_ exposure for 3 months (Fig. 6E). Overall, these results showed that UCB-Exo downregulated TGF-β/ID1 signaling and their therapeutic antifibrotic effect was superior to that of PB-Exo.

## Discussion

**Fig. 7:**
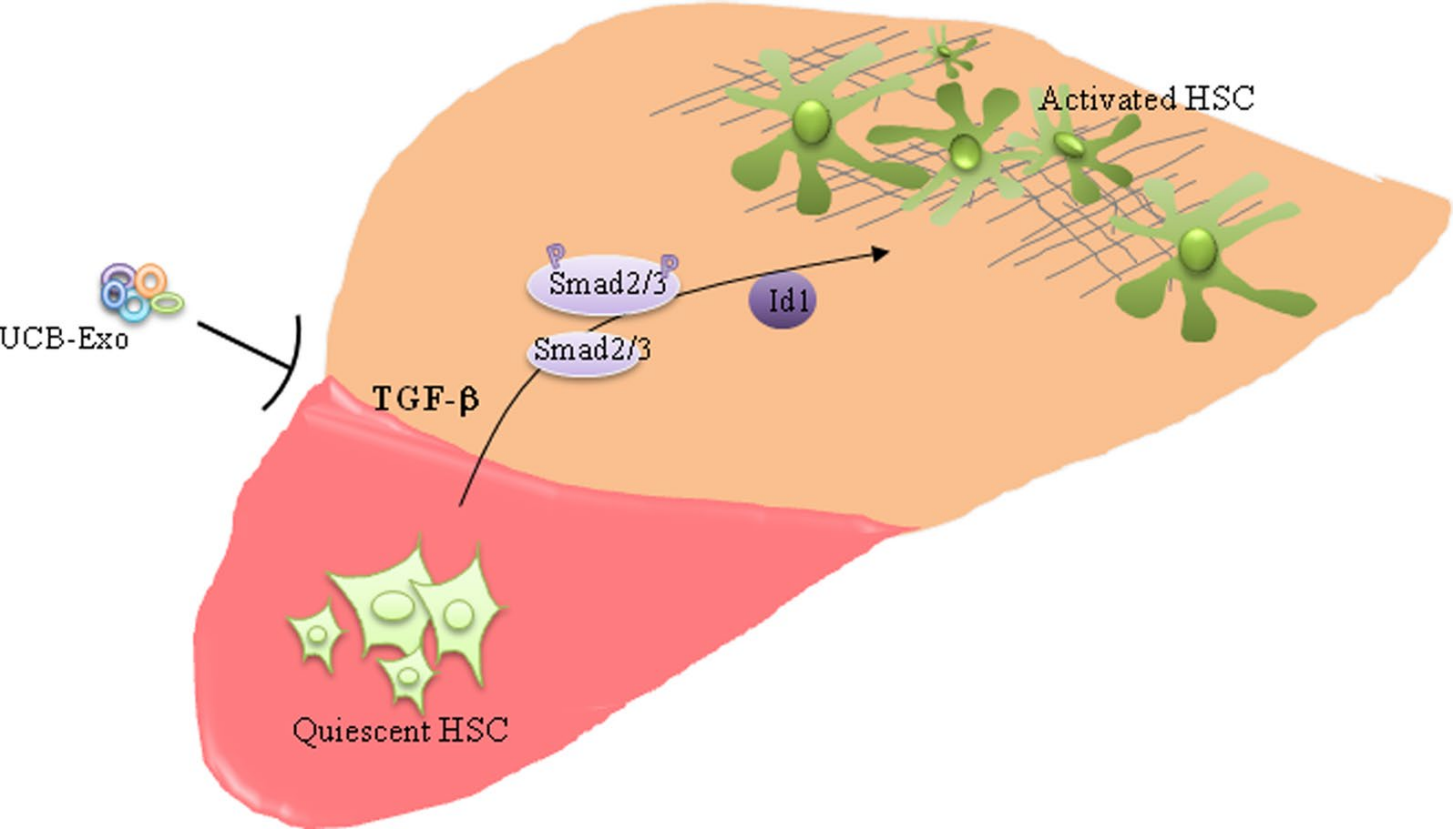
The mechanism of the antifibrotic effect of UCB-Exo; exosomes derived from UCB could suppress the TGF-β/ID1 signaling pathway, thereby inhibiting liver fibrosis

Liver fibrosis is irreversible and this passive process leads to progressive cirrhosis and liver failure. However, the molecular and cellular mechanisms of liver fibrosis continue to be developed. Currently, no specific antifibrotic options are available to prevent or cure liver cirrhosis. Thus, it is necessary to develop a safe, effective, and clinically viable strategy for prevention of hepatic fibrogenesis and improvement of hepatic function. Recent reports described that exosomes may provide a therapeutic opportunity, due to them playing important roles in paracrine and autocrine signaling [30]. In this study, antifibrotic effects of UCB-Exo and the potential mechanisms were identified. Liver fibrosis in the mouse model was induced by CCl_4_ administration. Histopathological and molecular biological changes started at 2 months and continued to develop. It was shown that eight infusions of UCB-Exo mitigated some of these changes. Additionally, UCB-Exo inhibited HSC proliferation, and increased MMP/TIMP degradation to reduce collagen production, mediated via downregulation of the TGF-β-ID1 signaling pathway (Fig. 7). Our findings may underpin future antifibrotic therapies to prevent the progression of liver fibrosis and improve liver function.

Exosomes are considered to enhance or improve liver function and regeneration [31, 32]. They act as messengers of intercellular communication and regulate the biological properties of recipient cells [33–35]. Exosomes originate from various types of cells and carry different therapeutic values. Currently, a major source of exosomes is MSCs, found in bone marrow, umbilical cord blood, and adipose tissue [36, 37]. They are able to attenuate liver fibrosis [38]. Compared to cell-based therapies, exosomes are powerful carriers that move across biological barriers [39, 40]. In addition, no immune response has been reported in patients undergoing MSC-Exo therapy [41]. Further advantages of exosome therapy include reduction of tumor risk and ease of storage, with the literature suggesting that MSC-Exo inhibit tumor growth [42, 43]. Even though exosome therapy possesses multiple advantages over cell-based therapies, there still exist some challenges. Before application of MSC-Exo, MSCs must be acquired and incubated, and the quality of cells and exosomes are affected by the period of culture. Therefore, using the exosomes derived from UCB plasma could avoid the time required for the culture of MSCs and be utilized immediately. In physiology, UCB plasma regulates the proliferation and function of MSCs, and represents a reservoir of growth factors, cytokines, and immunomodulatory mediators, whose concentrations are greater than that in MSC condition media [14]. Moreover, UCB plasma can be a FBS replacement for culturing MSCs [44], and may be available from a large number of high-quality cord blood banks worldwide. Hence, it may represent a novel compelling cell-free therapeutic option. In the present study, we identified that exosomes exist in UCB plasma and possess antifibrotic properties in diminishing collagen deposition in liver fibrogenesis. UCB plasma has been shown to improve functional performance and reduce structural damage in ischemic brain injury in rats [16]. Another study showed that UCB plasma improved hippocampal function in aged mice [45]. Previous findings also suggested that UCB plasma enhances liver function and reduces inflammation in rats with acute liver failure. In the present study, our results demonstrated that exosomes are present in both UCB and PB plasma. Nevertheless, bioinformatic analysis showed that their protein compositions differ. In vitro cell proliferation assay showed that UCB-Exo could inhibit HSC proliferation, in contrast to PB-Exo, which instead increased HSC proliferation. Therefore, UCB-Exo exerted more desirable effects in liver fibrosis.

TGF-β is a crucial mediator for HSC activation and ECM accumulation in liver fibrogenesis [46, 47]. In our study, TGF-β expression was reduced in mice with liver fibrosis treated with UCB-Exo, suggesting that the antifibrotic effects of UCB-Exo may be mediated through the suppression of TGF-β. In addition, TGF-β is a profibrotic factor that increases α-SMA expression and collagen I production in HSCs [4, 48]. UCB-Exo treatment could diminish α-SMA expression, and cell proliferation was reduced with in vitro TGF-β stimulation, suggesting that UCB-Exo decreased HSC activation in vitro and in vivo. Moreover, TGF-β signaling induces liver fibrosis through the sequential activation of downstream mediators including Smad2 and Smad3 [46, 47]. In this study, phospho-Smad2/3 was significantly increased after TGF-β stimulation. UCB-Exo pretreatment inhibited the expression of phospho-Smad2/3 after TGF-β stimulation. Furthermore, ID1 is one of the downstream effectors in TGF-β signaling and participates in cell differentiation and the cell cycle. Several reports have provided evidence that ID1 expression is associated with TGF-β expression [49–51]. Our results demonstrated that ID1 expression was significantly increased after TGF-β stimulation, but more importantly, UCB-Exo pretreatment was able to suppress this effect while PB-Exo pretreatment could not. Thus, our study highlights a key role for UCB-Exo in antifibrotic applications for liver fibrosis. A weakness of our study is that we did not collect short-term evaluation data after UCB-Exo treatment, and we did not use surface marker analysis to clearly identify the exosomes. The other limitation of this study is that proteomic analysis of exosomes cannot exclude the possibility of contamination from unexpected molecules pulled down together during extraction.

In conclusion, our findings suggest that UCB-Exo ameliorate CCl_4_-induced liver fibrosis. UCB-Exo therapy significantly decreases collagen production under both in vitro and in vivo conditions. Inhibition of HSC proliferation and subsequent events reduce liver fibrosis. Moreover, UCB-Exo treatment can mitigate liver fibrosis through inhibition of the TGF-β/ID1 signaling pathway. This study provides a novel mechanism for UCB-Exo regulated tissue repair and highlights an alternative source of exosomes in antifibrotic applications.

## Data Availability

All data generated or analyzed during this study are included in this published article.

## Abbreviations

UCB: Umbilical cord blood
PB: Peripheral blood
CCl_4_: Carbon tetrachloride
Exo: Exosome
MMP: Metalloproteinase
TIMP: Tissue inhibitor of metalloproteinase
HSC: Hepatic stellate cell
TGF-β: Transforming growth factor-β
ID1: Inhibitor of DNA binding 1
ECM: Extracellular matrix
d-GalN: d-galactosamine
AST: Aspartate aminotransferase
ALT: Alanine aminotransferase
